# The Regulation of the Hippo Signalling Pathway Effector YAP Through a Novel Lipid-Dependent Extracellular Matrix Complex

**DOI:** 10.3390/cells14211701

**Published:** 2025-10-30

**Authors:** Simge Karagil, Natalia Haddad, Michael Stolinski, Natasha Hill, Darren Johnson, Nadine Wehida, Ahmed Elbediwy

**Affiliations:** 1 Department of Biomolecular Sciences, Kingston University London, Kingston-upon-Thames KT1 2EE, UK; 2 Department of Molecular Biology and Genetics, Faculty of Arts and Sciences, Cyprus International University (CIU), Via Mersin 10, 99258 Nicosia, Turkey; 3 School of Biomedical Sciences, Portland Square, Plymouth PL4 8AA, UK

**Keywords:** cancer, hippo signalling pathway, YAP, lipid metabolism, extracellular matrix

## Abstract

Lipid metabolism plays a significant role in the regulation of various critical pathways within cells, where enhanced lipid metabolism is a hallmark of cancer cell metabolism. The Hippo signalling pathway poses as an important signalling pathway that governs tissue growth control and tumorigenesis. The effector of the Hippo signalling pathway, Yes-associated protein (YAP), serves as a central regulator for this growth control. Once a tissue develops to its correct size, YAP is phosphorylated and inactivated within the cytoplasm by the activation of the Hippo pathway, where its inactivation results in YAP nucleus translocation. This allows its dephosphorylation, modulating various cellular behaviours such as cellular proliferation and the inhibition of apoptosis. Moreover, it has been established that YAP is positively regulated by the extracellular matrix (ECM). The interplay between the Hippo signalling pathway, the ECM, and lipid metabolism, however, is not entirely clear. Thus, this study illustrates a novel link between the Hippo signalling pathway, the ECM, and lipid metabolism. Furthermore, the project identifies a novel ECM complex which is dependent upon lipids and regulates YAP in a positive manner.

## 1. Introduction

Lipid metabolism is a key cellular process which governs several biochemical, anabolic, and catabolic processes involving storage and structural integrity. In addition to these critical roles, lipids also control cellular signalling. Lipid rafts, for instance, have been linked to the compartmentalization of signalling pathways [1], while lipids are known to encourage cancer progression and metastasis [2,3]. The growth and proliferation of cancer cells require lipids for structure and for fuel to support proliferation, survival, migration, invasion, and metastasis [4,5]. On the other hand, the process of cancer invasion tends to degrade the surrounding ECM.

The ECM plays a critical role in cancer by providing cellular support and basal adhesion, and is a central point for the regulation of a range of intracellular signalling pathways [6]. Cancer cells are physically connected and sense the ECM through integrins and focal adhesions (FAs). Cells act as stress sensors and external mechanical cues are transmitted from FAs and converted to biochemical relays through integrin-related signalling pathways encouraging proliferation, migration, and survival [7].

The ECM primarily comprises integrin adhesion to the basement membrane of the cell, with other proteins such as talin and actinin activated in response to the mechanical stress that is exerted by the cell [8]. Talin is a protein which consists of a globular head domain and a rod domain. Talin directly binds to integrins and regulates integrin signalling. The mechanical stresses of cancer cells change the conformation of talin and enhance signalling pathways and interactions either positively or negatively. Talin is known to enhance various ECM-based signalling pathways such as Src family tyrosine kinases. Importantly, one of the key targets of ECM signalling and a pro-cancer protein is Yes-associated protein (YAP).

YAP is a mechanical sensor of the cellular microenvironment that can be regulated by several factors such as soluble extracellular factors, cell-to-cell adhesion, and mechanotransduction. It also plays a central role in delivering mechanical cues from surrounding cells to the transcriptional machinery of the nucleus [9,10]. YAP activation can vary, and the protein tends to shuttle between the cytoplasm and nucleus to induce the expression of cell-proliferative and anti-apoptotic genes by interacting with transcription factors, especially the transcriptional enhanced associated domain (TEAD) family members [11]. When a tissue reaches a certain size, the Hippo signalling pathway and its core kinases phosphorylate via Lats1/2 YAP, sequestering the protein in the cytoplasm. The type of substrate and the resulting mechanical tension are the key determinants of whether YAP is active or inactive. The mechanotransduction of a cancer cell affects YAP through the canonical Hippo signalling pathway as well as through ECM stiffness. Src tyrosine kinase is a known activator of YAP via ECM mechanotransduction, and a variety of other ECM proteins are known to react to mechanotransduction (FAK, vinculin, and talin control mechanosensitive YAP nuclear localization) and thus the knockdown of some of these ECM related proteins results in YAP becoming inactive and cytoplasmic [12]. Lipid metabolism also seems to regulate both the ECM and YAP. Some studies have suggested that fatty acids may enhance YAP activation [13]; however, the effect on YAP activation via lipogenesis has not been established yet. It has been identified that lipids such as phosphatidylinositol (4,5)-bisphosphate (PIP2) can activate ECM components such as talin and integrins [14], while the ECM itself can regulate lipids through proteins such as Lipin1 and SREBP1 [15]. However, the link between the ECM, the Hippo pathway, and lipids is currently unknown.

Oleic acid, a major monounsaturated fatty acid found in plant oils like olive oil as well as certain animal fats such as beef, has been found to exhibit both pro- and anticarcinogenic effects in various studies [16,17,18]. Oleic acid is a precursor to oleoylethanolamide (OEA), a lipid messenger involved in satiety. Another fatty acid of interest in this study is palmitic acid, which has also presented conflicting findings on its effect on cancer cells, with some literature studies deeming it a promoter of metastasis while others demonstrating that it exerts anti-tumour effects [19,20,21,22]. Palmitic acid can form lipokines like palmitoleic acid and fatty acyl esters of hydroxy fatty acids (FAHFAs), which may have anti-inflammatory and anti-diabetic effects [23].

This research aims to examine the effect of both oleic and palmitic acids on colorectal cancer cells, with an emphasis on how they interact with the Hippo signalling pathway where YAP serves as a central effector.

## 2. Materials and Methods

### 2.1. Human Cell Culture

Human Caco-2 cells (adenocarcinoma colon cells) were grown in Dulbecco’s Modified Eagles Medium (DMEM) supplemented with 10% heat-inactivated foetal calf serum (FCS), 100 µg/mL streptomycin and 100 µg/mL penicillin (Thermo Fisher Scientific, Loughborough, UK), in an incubator at 37 °C with a 5% CO_2_ atmosphere. All cells were subject to mycoplasma testing. Cells were plated in various types of tissue culture plates (6-well, 12-well, 24-well, 48-well and 96-well plates) at varying numbers of cells depending on the assay.

### 2.2. Scratch Assay

Cells were seeded at a density of 250k cells per well in 6-well plates (Thermo Fisher Scientific, Loughborough, UK), and left for two days to become confluent. Upon confluence, the cellular monolayer was scratched using the edge of a p200 pipette tip and washed once with PBS to remove cellular debris before being treated with 2 mM oleic and 0.5 mM palmitic acid. Pictures were taken at different timepoints (0 h, 24 h, and 48 h) using a brightfield microscope connected to an imaging tablet to calculate the rate of cellular migration. This was repeated 4 times (n = 4).

### 2.3. Co-Immunoprecipitation

Caco-2 cells were plated in 6-well plates at 100k. The cells were left for 48 h before being lysed for non-treated co-IPs or treated at 24 h with lipids and left for an additional 24 h before being lysed. Three 6-wells were combined for each co-IP, and before lysing were washed with ice-cold PBS. The cells were lysed using 0.5% Triton X100 in PBS, and homogenized with a 19-gauge needle and syringe. Lysates were then subjected to co-immunoprecipitation using magnetic beads (MCE^®^ MedChemExpress Protein A/G Magnetic Beads 1 mL Cat. #HY-K0202 Lot# 114185) (Cambridge Bioscience, Cambridge, UK). The lysis buffer composition was as follows (0.5% Tritonx100 in PBS). The lysis buffer was supplemented with Sigma Aldrich, Poole, UK P0044-1 ML Phosphatase Inhibitor Cocktail and Sigma Aldrich, Poole, UK P2714-1BTL Protease Inhibitor Cocktail Powder. Samples were left on ice to solubilize for 10 min, before being centrifuged (10,000 r.p.m. for 10 min at 4 °C), pre-cleared, and incubated with the magnetic beads for 2 h. IPs were subjected to three washes before being lysed in 2× sample buffer and boiled. For mass spectrometry, proteins were subjected to SDS-PAGE followed by Coomassie stain for 1 h and destained overnight. The bands of interest were cut and sent to the mass spectroscopy facility at the University of Birmingham. Antibodies used were at a concentration of 5 μg and bound to magnetic beads.

### 2.4. RNA Sequencing

Caco-2 cells were plated at 200k in a 6-well plate before being left for 24 h. The cells were then treated with 2 mM of oleic acid before being incubated for 24 h at 37 °C. After incubation, cell samples collating 3 independent experiments were pooled together, snap frozen using liquid nitrogen, and subsequently sent to GENEWIZ for RNA sequencing analysis on dry ice. The data was aligned and processed by GENWIZ and sorted by log2FoldChange, *p*-value, and *p*-value adjusted to show which genes were affected by the lipid treatment in comparison to the control.

RNA samples were quantified using Qubit 4.0 Fluorometer (Life Technologies, Carlsbad, CA, USA) and RNA integrity was checked with RNA Kit on Agilent 5300 Fragment Analyzer (Agilent Technologies, Palo Alto, CA, USA). ERCC RNA Spike-In Mix kit (cat. 4456740) from ThermoFisher Scientific, was added to normalized total RNA prior to library preparation following the manufacturer’s protocol. RNA sequencing libraries were prepared using the NEBNext Ultra II RNA Library Prep Kit for Illumina following the manufacturer’s instructions (NEB, Ipswich, MA, USA). Briefly, mRNAs were first enriched with Oligo(dT) beads. Enriched mRNAs were fragmented for 15 min at 94 °C. First strand and second strand cDNAs were subsequently synthesized. cDNA fragments were end repaired and adenylated at the 3′ ends, and universal adapters were ligated to cDNA fragments, followed by index addition and library enrichment by limited-cycle PCR. Sequencing libraries were validated using the NGS Kit on the Agilent 5300 Fragment Analyzer (Agilent Technologies, Palo Alto, CA, USA), and quantified by using a Qubit 4.0 Fluorometer (Invitrogen, Carlsbad, CA, USA). The sequencing libraries were multiplexed and loaded on the flowcell on the Illumina NovaSeq 6000 instrument according to manufacturer’s instructions. The samples were sequenced using a 2 × 150 Pair-End (PE) configuration v1.5. Image analysis and base calling were conducted by the NovaSeq Control Software v1.7 on the NovaSeq instrument (Illumina, Cambridge, UK). Raw sequence data (.bcl files) generated from Illumina NovaSeq was converted into fastq files and de-multiplexed using Illumina bcl2fastq program version 2.20. One mismatch was allowed for index sequence identification.

### 2.5. Lipid Staining

siRNA treatment was carried out following the siRNA transfection protocol shown below in the siRNA methodology. After 72 h of transfection, samples were subsequently treated with 1 mM oleic acid and incubated for a further 24 h. After 24 h, 1 µL of LipidSpotTM 488 Lipid Droplet Stain (Biotium, Cat:70065-T, Cambridge Bioscience, Cambridge, UK) and 0.25 µL of the nucleus stain DAPI were added into all wells and incubated for 30 min, before live cell images were taken using an EVOS fluorescent microscope (Thermo Fisher Scientific, Loughborough, UK).

### 2.6. Antibodies

Antibodies used in all experiments were rabbit anti-PDLIM7 (Novus, UK NBP1-84841), rabbit anti-YAP H-125 (Cat.# sc-15407), mouse anti-YAP (63.7) (Cat.# sc-101199) (both Santa Cruz Biotechnology (Distributed by Insight Biotechnology, Welwyn Garden City, UK), rabbit phospho-YAP (Ser127) (CST, UK, Cat no.#4911) CD2AP (B-4), mouse monoclonal (Cat.# sc-25272), mouse anti-Talin T3287-0.2 ml (SLS, UK), mouse ezrin antibody (3C12) (Cat.#sc-58758), and mouse GAPDH antibody (6C5) (Cat.#sc-32233) Santa Cruz Biotechnology (Distributed by Insight Biotechnology, Welwyn Garden City, UK).

### 2.7. Fixation and Lysing of Cells

Sterile coverslips were placed into 48-well plates using high-precision tweezers (Thermo Fisher Scientific, Loughborough, UK). CaCo-2 cells were seeded at varying densities (1:10, 1:15, 1:30, or 1:60) and incubated at 37 °C with 5% CO_2_ until reaching the desired confluency. Cells were fixed with 4% paraformaldehyde (Thermo Scientific) for 20 min in a fume cupboard and blocked with glycine/BSA/PBS buffer for 30 min or stored at 4 °C. Cells were lysed in 2× sample buffer (Tris-glycine SDS containing 1× sample reducing agent; Novex Thermo Fisher Scientific, Loughborough, UK).

### 2.8. siRNA Transfection

Cells were plated in 6-well plates at 1 × 10^5^ cells per well and immediately siRNA was transfected using Lipofectamine RNAiMax (Invitrogen, Paisley, UK) in Optimem and antibiotic-free medium (Thermo Scientific, UK) according to the manufacturer’s recommended protocol and as previously described [24]. Cells were transfected using a final concentration of 80 nM siRNA. Cells were left for a total of 72 h before being washed three times with PBS before either being fixed in paraformaldehyde for immunofluorescence or lysed in sample buffer for immunoblotting. Oligonucleotides used for YAP, PDLIM7, Talin, CD2AP and NC control were as a siGenome pool (Dharmacon, Cambridge, UK).

### 2.9. Lipid Treatments

For lipid experiments, cells were treated with the indicated concentrations and left for 24 h. For oleic acid this was 2 mM and for palmitic acid this was 0.5 mM. DMSO was used as a control. Oleic acid was as an oleic acid-albumin solution (Merck, London, UK); palmitic acid was from Cayman Chemicals (Distributed by Cambridge Bioscience, Cambridge, UK).

### 2.10. Microscopy

Images were taken on a Zeiss confocal microscope for siRNA experiments (63× oil immersion lens) and an EVOS epifluorescence microscope for brightfield images 4× or 10× lens.

### 2.11. SDS-PAGE

Protein separation was performed using sodium dodecyl sulfate/polyacrylamide gel electrophoresis (SDS-PAGE). A 10% polyacrylamide separating gel was prepared and cast into a Bio-Rad gel cassette between 1.5 mm glass plates. Gel cassettes were assembled in an electrophoresis tank and filled with 1× running buffer comprising Tris/Glycine/SDS.

Each lane was loaded with a molecular weight marker (Thermo Scientific, Loughborough, UK) and prepared protein samples. Electrophoresis was conducted at 180 V for 1 h using a Bio-Rad PowerPac system. Following electrophoresis, gels were processed for Western blotting.

### 2.12. Western Blotting

Following SDS-PAGE, proteins were transferred onto 0.45 μm nitrocellulose membranes (Sigma Aldrich, Poole, UK) using the Bio-Rad Trans-Blot system. Transfer was performed in 1× transfer buffer (Tris/Glycine/SDS and methanol) at 100 V for 90 min using a standard sandwich configuration.

Membranes were briefly rinsed with distilled water and stained with Amido Black for 20 s to visualize total protein content. Destaining was performed twice using destain solution on a shaker for 10 min each, followed by a 5 min wash in PBS to neutralize the membrane.

To block non-specific binding, membranes were incubated in 5% non-fat dry milk in PBS-T for 15 min. Primary antibody incubation was carried out overnight at 4 °C on a Stuart see-saw rocker. Membranes were then washed three times with PBS-T (10 min each) and incubated with LI-COR infrared secondary antibodies in 10 mL PBS-T for 90 min at room temperature in the dark.

After secondary incubation, membranes were washed three times with PBS-T and twice with PBS (10 min each) to remove residual detergent. The imaging and quantification of total protein levels were performed using the LI-COR Biosciences Western Blot Scanner (LI-COR Biosciences UK Ltd., Cambridge, UK), and normalization graphs were generated using the associated software.

### 2.13. Planaria Experiments

Two species of planarian, *Dugesia lugubris* (*D. lugubris*) and *Schmidtea mediterranea* (*S.med*), were used for microinjection experiments. Worms were immobilized on ice-covered Petri dishes layered with blue roll and screened under a Motic AE31 dissecting microscope for morphological abnormalities prior to injection. Oleic acid and palmitic acid were administered via micro-syringe (1 μL) into the tail region. Post-injection, the worms were transferred to tanks containing planarian water (1 g/L ocean salts and Bioactive Tapsafe) and monitored for physical changes at 1 and 2 weeks. Images were captured before and after injection.

For *S. mediterranea*, oleic and palmitic acid-treated worms were compared to controls to assess expression of Hippo and Yorkie genes via qPCR. Wound resolution was evaluated in oleic acid-treated worms relative to controls. In *D. lugubris*, whole worms and regenerating trunk fragments were compared following oleic acid treatment. Worms were sectioned into head, trunk, and tail using sterile scalpels, and trunk fragments were injected and monitored similarly.

Total RNA was extracted using RNAzol reagent (Merck, London, UK).Worms were homogenized in 500 μL RNAzol, followed by addition of 500 μL isopropanol, incubation at room temperature for 10 min, and centrifugation at 12,000× *g* for 10 min. The RNA pellet was washed twice with 75% ethanol, centrifuged at 8000× *g* for 2 min, and dissolved in 200 μL RNase-free water. RNA concentration was measured using NanoVue Plus™ (Merck, London, UK). and stored at −20 °C.

cDNA synthesis was performed using iScript RT Supermix (Bio-Rad, Oxford, UK) on a ProFlex PCR System. cDNA concentrations were measured and stored at −20 °C. Quantitative PCR was conducted using Eco PCRmax system.

### 2.14. Protein Identifications by LC-MS/MS Analysis

After gel bands were excised from SDS PAGE, they were destained and digested by a robot system. Briefly, Coomassie blue were destained with acetonitrile followed by 100 mM ammonium bicarbonate. This cycle was repeated if necessary until gel pieces were fully destained. Destained gel pieces were dried (vacuum centrifugation; 5 min) and rehydrated in 100 ammonium bicarbonate. Then the gel pieces were reduced with 10 mM DTT, at 60 °C for 15 min, and the liquid was removed and replaced with 50 mM iodoacetamide and 100 ammonium bicarbonate for alkylation. Gel pieces were incubated at room temperature in the dark for 45 min, washed with 100 ammonium bicarbonate and then dried for 5 min. Trypsin gold (Promega, Madison, WI, USA) solution was added in 100:1 ratio, shaken at room temperature for 20 min, before diluted with 100 mM ammonium bicarbonate. Hydrolysis was allowed to occur overnight (~16 h) at 37 °C. The gel pieces were first extracted with the solution of 2% acetonitrile and 0.1% formic acid in water and shaken for 30 min at room temperature. The remaining peptides in the gel were extracted by using 70% acetonitrile with 0.1% formic acid in water shaken for 30 min at room temperature. The supernatant was pooled and dried in an evaporator. The samples were re-suspended in 0.1% formic acid in water for the LC-MS/MS analysis.

### 2.15. LC-MS/MS Experiment

UltiMate^®^ 3000 HPLC series (Dionex, Sunnyvale, CA, USA) was used for peptide concentration and separation. Samples were trapped on uPrecolumn Cartridge, Acclaim PepMap 100 C18, 5 um, 100 A 300 um i.d. × 5 mm (Dionex, Sunnyvale, CA, USA) and separated in Nano Series™ Standard Columns 75 µm i.d. × 15 cm, packed with C18 PepMap100, 2 µm, 100 Å (Dionex, Sunnyvale, CA, USA). The gradient used was from 3.2% to 44% solvent B (0.1% formic acid in acetonitrile) for 30 min. Peptides were eluted directly (~350 nL min^−1^) via a Triversa Nanomate nanospray source (Advion Biosciences, New York, NY, USA) into a QExactive HF (QEHF) mass spectrometer (Thermo Fisher Scientific, Loughborough, UK). The data-dependent scanning acquisition is controlled by Xcalibur 4.0 software. The mass spectrometer alternated between a full FT-MS scan (m/z 360–1600) and subsequent high-energy collision dissociation (HCD) MS/MS scans of the 20 most abundant ions. Survey scans were acquired in the QEHF with a resolution of 120,000 at m/z 200 and automatic gain control (AGC) 3 × 10^6^. Precursor ions were fragmented in HCD MS/MS with resolution set up at 15,000 and a normalized collision energy of 28. ACG target for HCD MS/MS was 1 × 10^5^. The width of the precursor isolation window was 1.2 m/z and only multiply charged precursor ions were selected for MS/MS. Spectra were acquired for 56 min with dynamic exclusion time of 20 s. The MS and MS/MS scans are searched against Uniprot database using Proteome Discoverer 2.2 (ThermoFisher Scientific, UK). Variable modifications are deamidation (N and Q), oxidation (M) and phosphorylation (S, T and Y). The precursor mass tolerance is 10 ppm and the MS/MS mass tolerance was 0.02 Da. Two missed cleavages were allowed and were accepted as a real hit protein with at least two high confidence peptides.

### 2.16. Statistical Analysis

All statistical data was demonstrated using mean for the bars and standard deviation (SD) for the error bars. Statistical significance was defined by a Student’s t-test (n.s = not significant (*p* > 0.05), * *p* ≤ 0.05, ** *p* ≤ 0.01, and *** *p* ≤ 0.001). The representative N-values are indicated in figure legends. Statistical significance of the planarian qPCR data was assessed using a two-way ANOVA, followed by Bonferroni’s post hoc test to compare treatment groups with controls. Differences were considered statistically significant at *p* < 0.05 for comparisons involving oleic or palmitic acid-treated planarians versus untreated controls. For wound resolution analysis in oleic acid-treated S. med, significance was determined using the Mann–Whitney U test, with *p* < 0.05 considered significant.

## 3. Results

### 3.1. Lipid Treatment Impairs Colorectal Cancer Cell Migration and Modulates YAP Activity via the Hippo Pathway

**Figure 1:**
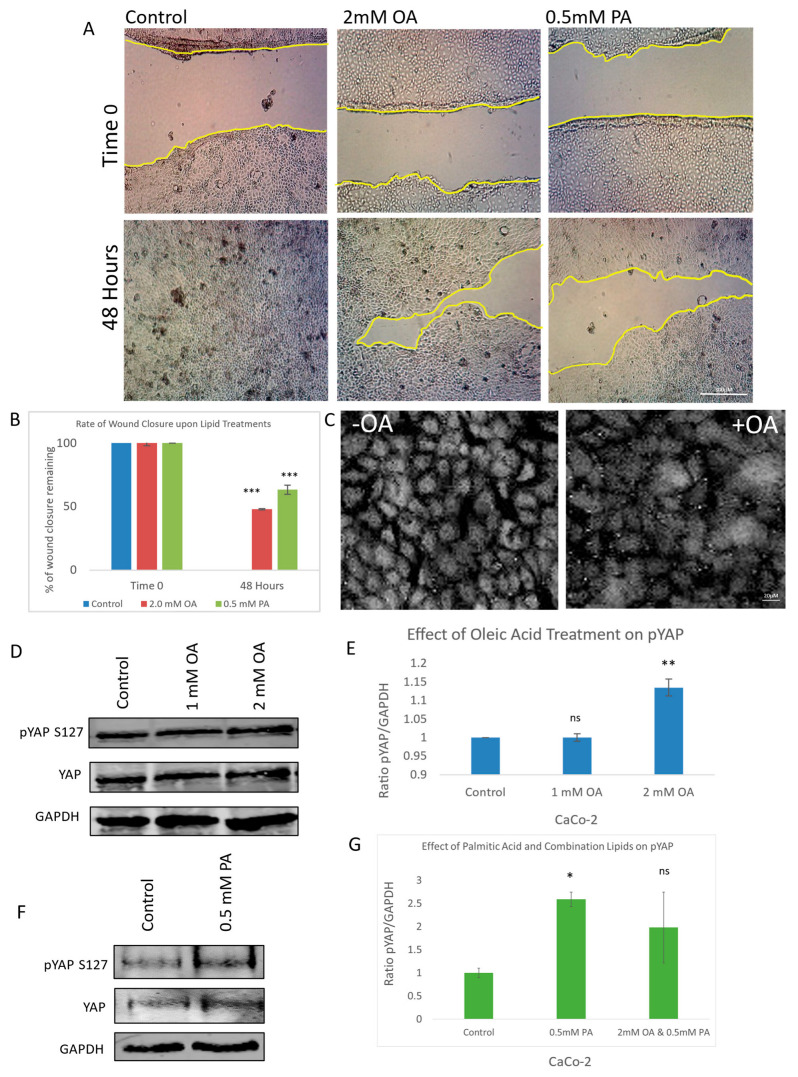
Effect of oleic and palmitic acid on YAP phosphorylation, migration, and translocation. (**A**) Representative brightfield images of a scratch wound (migration assay) showing the effect of oleic acid (OA) and palmitic acid (PA) at 0 and 48 h post-scratch compared to control. (**B**) Graph illustrating the rate of wound closure upon OA and PA treatment in comparison to control. (**C**) Immunofluorescent images of YAP with and without OA treatment observing the amount of nuclear YAP present. (**D**) Immunoblots illustrating the effect of OA treatment on pYAP (S127), total YAP, and a housekeeping gene (GAPDH). (**E**) Graph illustrating the ratio between pYAP and housekeeping gene when treated with OA in comparison to control. (**F**) Immunoblots illustrating the effect of PA treatment on pYAP (S127), total YAP, and a housekeeping gene (GAPDH). (**G**) Graph illustrating the ratio between pYAP and housekeeping gene when treated with OA, PA, or combination in comparison to control. Scale bar for (**A**) 300 μM; (**C**) 20 μM. Data in graphs are presented as mean ± SD. (* = *p* ≤ 0.05; ** = *p* ≤ 0.01, *** = *p* ≤ 0.001, ns = *p* > 0.05, not significant; Student’s T-Test). Experiments were repeated 3–4 times (n = 3/n = 4).

To investigate the impact of lipid exposure on cell migration, a scratch wound assay was performed using both oleic acid and palmitic acid at their optimal concentrations after initial testing using a range of concentrations. Quantitative analysis of wound closure over time revealed that lipid-treated cells exhibited significantly delayed cellular migration compared to the control (Figure 1A). This impairment in wound healing was statistically validated and visualized through graphical representation (Figure 1B), indicating that lipid treatment perturbs cellular motility.

To determine whether this anti-migratory effect was linked to modulation of the Hippo signalling pathway, we examined the subcellular localization of Yes-associated protein (YAP), a key downstream effector. Immunofluorescence staining of YAP revealed a marked reduction in nuclear YAP levels in oleic acid-treated cells relative to the control (Figure 1C), suggesting decreased transcriptional activity. To further confirm YAP inactivation, we assessed its phosphorylation status at serine 127—a known LATS kinase target site—using a phospho-specific antibody. Oleic acid treatment induced a dose-dependent increase in YAP phosphorylation, with statistically significant elevation observed at the 2 mM concentration (Figure 1D,E). This phosphorylation event is consistent with canonical Hippo pathway activation, leading to cytoplasmic retention and the functional inhibition of YAP.

Palmitic acid (PA) was similarly evaluated and found to increase YAP phosphorylation at S127, also exhibiting statistical significance (Figure 1F,G). Interestingly, co-treatment with both oleic and palmitic acids did not produce an additive effect on YAP phosphorylation (Figure 1G), suggesting that the two lipids may exert their regulatory influence through overlapping or distinct mechanisms. These findings collectively demonstrate that lipid exposure not only impairs cell migration but also modulates YAP activity through enhanced phosphorylation, implicating the Hippo signalling pathway in the observed anti-cancer effects.

### 3.2. Oleic Acid Modulates YAP Transcriptional Activity and Hippo Pathway Gene Expression

Building on the findings from the initial experiments and given prior evidence linking palmitic acid (PA) to Hippo pathway regulation [25], we chose to focus specifically on oleic acid (OA) as the primary lipid for further mechanistic investigation. Having established that OA treatment alters YAP subcellular localization and promotes its inactivation via phosphorylation, we next sought to determine whether OA influences the transcriptional output of YAP. To this end, RNA sequencing was performed on OA-treated cells to assess if changes occurred in Hippo-signalling-pathway-related genes using gene expression profiles.

**Figure 2:**
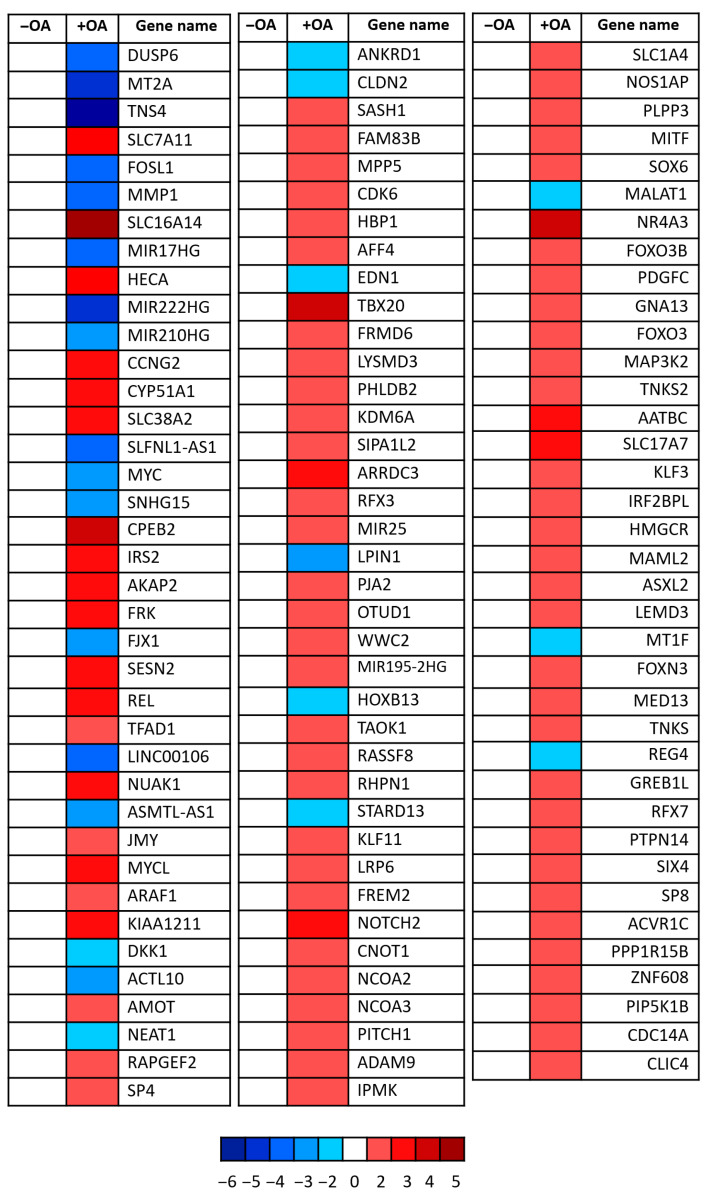
YAP target gene modulation upon oleic acid treatment. Heatmap showing relative expression changes in known YAP target genes following OA treatment. Each square represents a single gene, with red indicating upregulation and blue indicating downregulation relative to control conditions. Colour intensity corresponds to log_2_ fold-change values as indicated by the scale bar (ranging from −6 to +5). Data reflects transcriptional modulation of YAP-responsive genes involved in diverse biological processes including cell proliferation, metabolism, and cytoskeletal regulation. Three independent experiments were pooled.

Transcriptomic analysis revealed significant downregulation of several canonical YAP target genes following OA treatment (Figure 2. Notably, the proto-oncogene c-Myc and ANKRD1—both well-established transcriptional targets of YAP [24,26,27]—were clearly suppressed. In parallel, genes associated with upstream Hippo signalling pathway activation were upregulated, including AMOT, FRMD6, and WWC2, all of which are known to facilitate YAP inhibition through enhanced LATS kinase activity [28,29,30]. These findings strongly support the hypothesis that OA not only affects YAP localization and phosphorylation but also exerts transcriptional control over its downstream targets. Collectively, the data indicate that OA modulates the Hippo signalling cascade, positioning YAP as a central effector in the lipid-mediated regulation of cellular behaviour.

### 3.3. Lipid Regulation of YAP Is Mediated Through a Novel ECM-Associated Protein Complex

**Figure 3:**
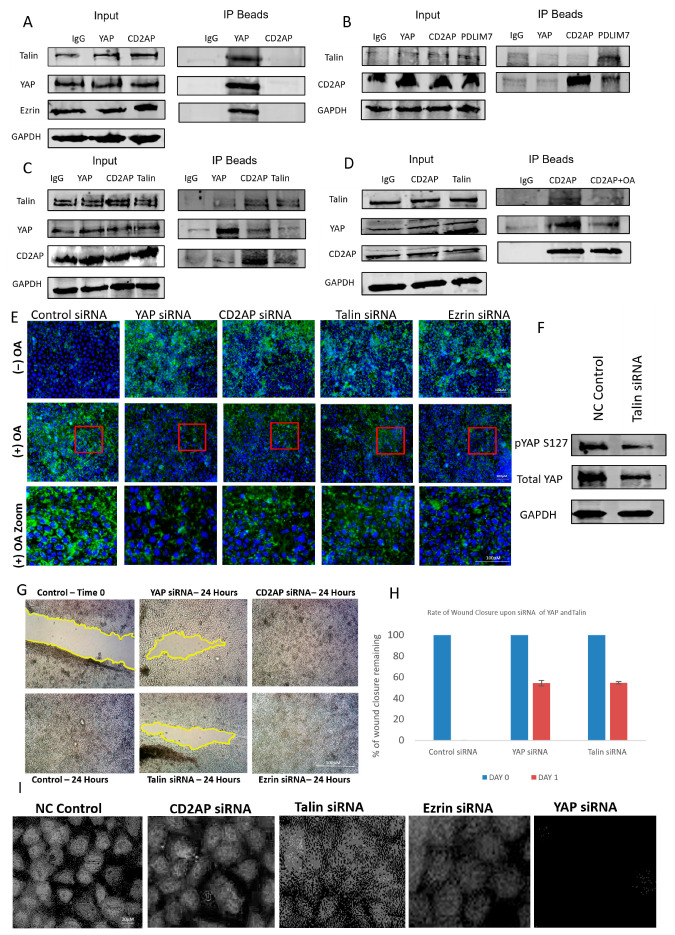
Characterization of a novel lipid-dependent YAP complex. (**A**–**C**) Western blots illustrating co-immunoprecipitations for Talin, CD2AP, YAP, PDLIM7, and Ezrin. GAPDH was used for assessing loading. (**D**) Western blots illustrating co-IPs for talin, CD2AP, YAP, PDLIM7, and ezrin using OA to disrupt the interactions of the complex. GAPDH was used for assessing loading. (**E**) Immunofluorescent images of YAP with and without OA treatment and different siRNA knockdowns of YAP, CD2AP, talin, and ezrin observing the amount of lipid droplets present in the cell. Red box illustrates zoomed in area. Scale bar 100 μm. (**F**) Immunoblots illustrating the effect of Talin siRNA on pYAP (S127), total YAP, and a housekeeping gene (GAPDH). (**G**) Representative brightfield images of a scratch wound (migration assay) showing effect of siRNA of YAP, talin, CD2AP, and ezrin at 0 and 48 h post-scratch compared to control. Scale bar 300 μm. (**H**) Graph illustrating the rate of wound closure upon siRNA of YAP, Talin, CD2AP, and ezrin in comparison to control. (**I**) Immunofluorescent images showing the effect of siRNA on YAP, talin, CD2AP, and ezrin and observing the amount of nuclear YAP present. Scale bar 20 μm. Experiments were repeated 3–4 times (n = 3/n = 4).

To further investigate the mechanism by which oleic acid regulates YAP activity, we explored the potential involvement of extracellular matrix (ECM) components in this process. Previous studies have implicated ECM proteins in modulating YAP localization and activity, and using a candidate-based approach supported by literature evidence, we focused on talin and PDLIM7—two proteins previously shown to interact with YAP [8]. To identify additional components of this regulatory axis, mass spectrometry analysis was performed, revealing novel YAP-associated proteins including CD2AP, a capping protein-associated molecule, and ezrin, a polarity-regulating protein (Figure S1). These interactions were validated through co-immunoprecipitation experiments, which confirmed the formation of a discrete protein complex comprising YAP, talin, CD2AP, and ezrin (Figure 3A–C).

To assess whether this complex was also regulated by lipids as was the case with YAP, cells were exposed to oleic acid (OA), and protein–protein interactions were re-evaluated. OA treatment disrupted the association between YAP and both CD2AP and talin, indicating that lipid exposure can destabilize this novel ECM-linked complex (Figure 3D). This suggests that lipid-mediated regulation of YAP may occur, at least in part, through interference with its binding partners within the ECM. To further explore the functional consequences of this disruption, we examined lipid droplet accumulation in cells following siRNA-mediated knockdown of individual complex components. Knockdown of CD2AP, ezrin, or talin led to increased intracellular lipid accumulation, whereas knockdown in OA-treated cells resulted in a significant reduction in lipid content (Figure 3E). These findings imply that the complex may play a role in lipid trafficking or metabolism, and its disruption by OA alters lipid homeostasis.

Interestingly, talin knockdown produced a paradoxical effect on YAP phosphorylation and localization. While phosphorylation of YAP was reduced, nuclear YAP levels also decreased (Figure 3F,I), suggesting that talin may regulate YAP through non-canonical mechanisms, possibly involving cytoskeletal tension or mechanical signalling. Knockdown of CD2AP and ezrin resulted in only modest reductions in nuclear YAP, reinforcing the unique role of talin in modulating YAP activity. These observations highlight the complexity of YAP regulation and suggest that talin may serve as a critical mediator linking ECM dynamics to Hippo pathway signalling.

To evaluate the functional relevance of this complex in cancer cell behaviour, we performed scratch wound assays following siRNA knockdown of each component. Knockdown of YAP and talin significantly impaired cell migration, whereas knockdown of CD2AP and ezrin had no observable effect (Figure 3G). This suggests that while CD2AP and ezrin may serve structural or scaffolding roles within the complex, the regulatory axis is primarily driven by the YAP–talin interaction. Together, these findings reveal a novel lipid-sensitive ECM-associated complex that modulates YAP activity and cell migration, providing new insights into the mechanotransduction and metabolic regulation of the Hippo signalling pathway.

### 3.4. Oleic Acid Impairs Regeneration and Modulates Hippo Pathway Gene Expression In Vivo

To determine whether the lipid-mediated effects observed in vitro could be recapitulated in a physiological context, we employed Schmidtea mediterranea (*S.med*) (planaria) as an in vivo model system. Planarians are widely recognised for their exceptional regenerative capacity and serve as a powerful model for studying stem cell behaviour, tissue remodelling, and conserved signalling pathways such as Wnt, Notch, and the Hippo signalling pathway. Their simple body plan, ease of manipulation, and well-characterized genome make them particularly suitable for investigating cellular responses to environmental and molecular cues, including lipid exposure.

**Figure 4:**
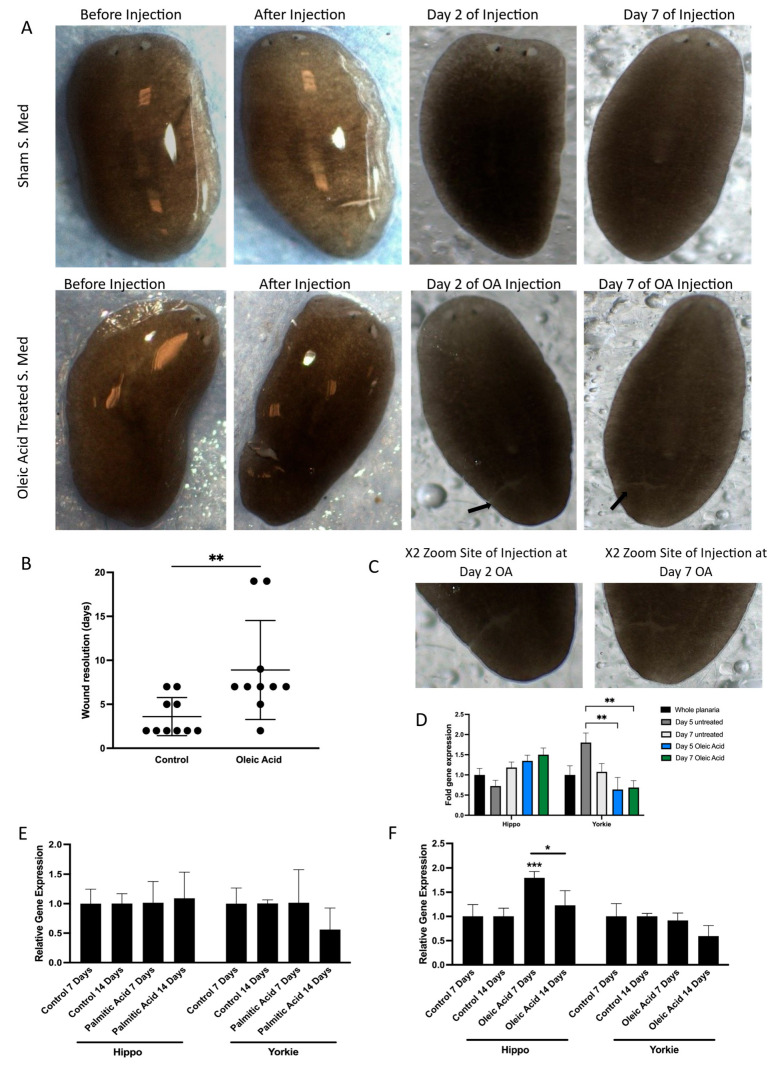
Effect of lipids on YAP modulation in vivo. (**A**) Images illustrated of *S.med* worms exhibiting wound resolution when treated with oleic acid and compared to control treated worms. Representative images of *S.med* before OA treatment and post microinjection. Wound resolution was presented at Day 2 and Day 7. Two arrows are indicative of sites of wounding for oleic acid-treated *S.med* at Day 2 and Day 7. (**B**) Scatter plot illustrates the different timing of wound resolution as days in OA-treated *S.med* when compared to control. Black dots represent *S.med* worms not wounded. n = 10. The *p*-value was calculated by Mann–Whitney U test, significant at ** *p* ≤ 0.01. (**C**) focused images show the site of injury for Day 2 and Day 7 oleic acid-treated *S.med* planaria. (**D**) Comparisons between Day 5 untreated versus Day 5 OA and Day 7 OA were performed using Bonferroni’s multiple comparisons test: ** *p* ≤ 0.01. Error bars represent ±SEM, n = 12. (**E**) *S.med* worms were injected with PA. The expression of Hippo and Yorkie genes was assessed using RT-qPCR at Day 7 and Day 14 post-treatment in comparison to control. ACBT was used as a housekeeping gene. Error bars represent ±SEM, n = 6. (**F**) *S.med* worms were injected with OA. The expression of Hippo and Yorkie genes was assessed using RT-qPCR at Day 7 and Day 14 post-treatment in comparison to Control. ACBT was used as a housekeeping gene. Error bars represent ±SEM, n = 6 Overall significant interaction was obtained by two-way ANOVA. Statistically significant increase was obtained in expression of Hippo gene in OA-treated *S.med* (*** *p* ≤ 0.001 and (* *p* ≤ 0.05). Error bars represent ±SEM, n = 6.

The initial aim was to assess whether oleic acid (OA) treatment would influence wound closure dynamics in vivo similarly to what was seen in vitro with the migration assays in Caco-2 cells. Following injury, planaria were exposed to OA, and wound healing was monitored over time. Compared to controls, OA-treated planaria exhibited a significant delay in wound closure (Figure 4A–C), closely mirroring the perturbation of migration seen in cultured cells. This result confirmed that OA also impairs regenerative processes in vivo, likely through conserved molecular mechanisms.

To investigate whether this impairment was linked to modulation of the Hippo signalling pathway, we analysed the expression of key pathway components. Specifically, we measured the transcription levels of both Hippo and Yorkie, the planarian orthologues of MST and YAP, respectively. Short-term OA exposure resulted in a significant downregulation of Yorkie expression (Figure 4D), suggesting that OA suppresses YAP activity at the transcriptional level in vivo. This finding aligns with our earlier observations of reduced nuclear YAP and increased phosphorylation in vitro, reinforcing the idea that OA acts as a negative regulator of YAP signalling.

To further explore the long-term effects of lipid exposure, planaria were treated with sustained doses of both OA and palmitic acid (PA). Interestingly, while PA had no measurable impact on gene expression, long-term OA treatment led to a significant upregulation of Hippo gene expression (Figure 4E,F). This suggests that prolonged exposure to OA may activate upstream components of the Hippo pathway, potentially enhancing the inhibitory signalling cascade that leads to YAP inactivation. The differential response between OA and PA also highlights the specificity of lipid signalling in modulating regenerative and transcriptional outcomes.

These findings demonstrate that OA disrupts wound healing and modulates Hippo pathway gene expression both in vitro and in vivo, validating the relevance of this model for studying lipid–signalling interactions in vivo.

**Figure 5:**
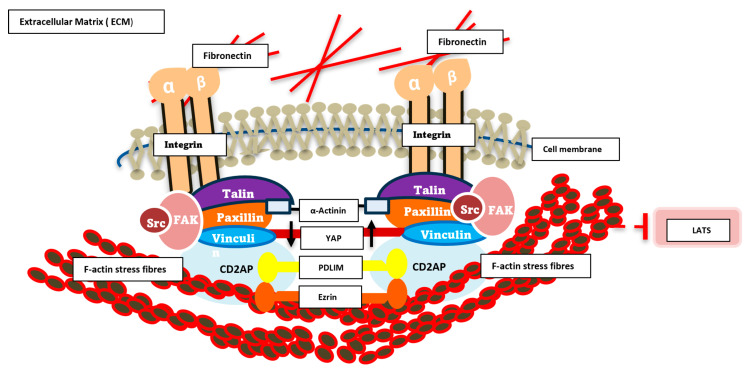
Schematic diagram of signalling dynamics in the ECM/cytoskeleton. This schematic illustrates the key molecular components involved in cell adhesion and mechanotransduction at focal adhesions. Integrin receptors (α and β subunits) span the plasma membrane, linking extracellular fibronectin within the extracellular matrix (ECM) to intracellular adaptor and signalling proteins. Talin, Paxillin, Vinculin, Src, and FAK form a dynamic protein complex that anchors integrins to the actin cytoskeleton, represented by F-actin stress fibres and crosslinking α-Actinin. These interactions facilitate mechanical force transmission and initiate downstream signalling cascades. Cytoplasmic signalling molecules, including the novel complex YAP, PDLM, CD2AP and ezrin, are shown participating in mechanosensitive pathways that regulate cellular responses such as migration, proliferation, and gene expression. The diagram highlights the integrative role of focal adhesions as hubs for biochemical and biomechanical signal transduction.

In order to bring this all together in some form of schematic to address how we hypothesize our novel ECM complex integrates with YAP and the cytoskeleton, we have tried to illustrate this in Figure 5.

## 4. Discussion

### 4.1. Lipid-Induced Modulation of YAP Activity in Colorectal Cancer

Lipid metabolism is increasingly recognised as a central regulator of cancer cellular behaviour. Cancer cells tend to reprogram their own metabolic pathways to support their rapid proliferation, for enhanced growth [31]. Lipids serve not only as structural components and energy sources but also as signalling molecules that modulate oncogenic pathways. Notably, fatty acid metabolism is upregulated in many cancers, contributing to tumour growth and resistance to therapy [32].

OA, a monounsaturated fatty acid, has been shown to influence cancer cell stemness and survival under glucose-deficient conditions by activating stearoyl-CoA desaturase (SCD), which in turn promotes YAP nuclear translocation [33].

In this study, we demonstrate that exposure to specific fatty acids, specifically oleic acid (OA) and palmitic acid (PA), modulates YAP activity in colorectal cancer cells, with distinct outcomes depending on the lipid species. OA treatment led to the increased phosphorylation of YAP at Serine 127, a key LATS1/2 target site, resulting in cytoplasmic retention and functional inactivation of the protein. This finding contrasts with previous reports in other cancer contexts, such as breast and liver cancer, where OA has been shown to promote YAP nuclear translocation and stemness under metabolic stress conditions [33,34]. These discrepancies highlight the context-dependent nature of lipid–YAP interactions and suggest that the regulatory effects of fatty acids may vary significantly depending on cell type, tissue origin, and microenvironmental cues. Stress is known to activate YAP in a variety of context [35], so this could be a key discrepancy.

Importantly, our results reveal a suppressive effect of OA on YAP-driven transcriptional activity, as evidenced by the downregulation of canonical YAP target genes including c-Myc and ANKRD1. In parallel, we observed the upregulation of upstream Hippo pathway regulators such as AMOT, FRMD6, and WWC2, which are known to reinforce YAP inhibition through enhanced LATS kinase activity [24]. These transcriptional changes were confirmed by RNA sequencing and support the hypothesis that lipid exposure can regulate Hippo pathway signalling in colorectal cancer.

While these findings provide novel insights into lipid YAP regulation, it is important to acknowledge the limitations of the study. The observed effects were characterized in specific colorectal cancer cell lines and may not be generalizable across other cancer types or normal epithelial cells. Additionally, the lipid concentrations used in vitro vary considerably from physiological levels typically found in human plasma or tissues. Although these doses were necessary to elicit measurable effects, future studies should aim to replicate these findings under more physiologically relevant conditions to better understand their translational potential.

### 4.2. Lipid-Sensitive Regulation of the Hippo Pathway

The Hippo signalling pathway is a conserved regulator of tissue growth, cell proliferation, and apoptosis. Its core kinases, MST1/2 and LATS1/2, phosphorylate YAP and TAZ, leading to their cytoplasmic sequestration and degradation. When Hippo pathway signalling is inactive, YAP translocates to the nucleus and drives the transcription of genes that promote cell growth and survival.

Our data show that both OA and PA increase YAP phosphorylation, thereby inhibiting its nuclear localization and transcriptional activity. This was confirmed through immunofluorescence and Western blot analysis, and further supported by transcriptomic profiling. These findings suggest that lipid exposure can activate Hippo pathway signalling and suppress YAP function, offering a potential strategy for targeting YAP-driven oncogenic transcription in colorectal cancer.

Interestingly, OA disrupted YAP’s interaction with key ECM-associated proteins, suggesting that OA may differentially regulate YAP through distinct mechanisms compared to PA. This observation warrants further investigation into the lipid-specific modulation of Hippo pathway components and their downstream effects.

### 4.3. Crosstalk Between ECM, Lipids, and YAP Signalling

To explore the mechanistic basis of lipid-induced YAP regulation, we investigated the role of the extracellular matrix (ECM) and its associated cytoskeletal and polarity proteins. The ECM is a dynamic structure that influences cell behaviour through mechanical and biochemical cues, and its stiffness and composition are known to regulate YAP activity via mechanotransduction pathways.

We identified a novel ECM-associated protein complex comprising talin, CD2AP, ezrin, and PDLIM7, all of which interact with YAP under basal conditions. Co-immunoprecipitation experiments confirmed the formation of this complex, and OA treatment disrupted these interactions, indicating that the complex is lipid-sensitive. Talin, a key mechanosensor, links integrins to the actin cytoskeleton and regulates focal adhesion dynamics. Mutations in talin have been shown to disrupt cell adhesion and migration, contributing to cancer progression [36]. Knockdown of talin reduced YAP phosphorylation but also decreased nuclear YAP, suggesting a dual role in both scaffolding and signalling. CD2AP and ezrin, known to associate with lipid rafts and regulate membrane dynamics, may serve as structural components facilitating lipid transport and ECM–cytoskeleton communication [37,38]. Ezrin has also been linked to membrane PIP2 binding and phosphorylation [39]. PDLIM7, which binds directly to YAP via its PDZ domain, likely contributes to YAP’s nuclear localization and transcriptional activity.

Functional assays revealed that knockdown of YAP and talin significantly impaired cell migration, while knockdown of CD2AP and ezrin had minimal effects, reinforcing the central role of the YAP–talin interaction in regulating cellular motility. These findings suggest that mechanical and lipid cues converge at the ECM/YAP interface to modulate cell behaviour, and that disruption of this complex may impair YAP function and migration.

### 4.4. Validation Using an In Vivo Model

To validate the physiological relevance of our findings, we employed *Schmidtea mediterranea* (*S.med*) (planaria) as an in vivo model system. Planaria are widely used for studying regeneration and stem cell dynamics due to their robust regenerative capacity and conserved signalling pathways. Their simplicity and ease of manipulation make them ideal for assessing the impact of lipid signalling on tissue remodelling.

OA treatment significantly delayed wound healing in planaria, mirroring the effect on cancer cell migration observed in vitro. Gene expression analysis revealed the downregulation of Yorkie (the planarian orthologue of YAP) and upregulation of Hippo (the MST orthologue), suggesting activation of the Hippo pathway and suppression of YAP activity in vivo. Interestingly, PA had no significant effect on gene expression, reinforcing the specificity of OA in modulating Hippo pathway signalling. These results confirm that lipid-mediated regulation of YAP is conserved across species and can influence regenerative outcomes in vivo.

### 4.5. Future Directions, Limitations, and Therapeutic Implications

The identification of a lipid-sensitive, YAP-containing novel ECM regulatory complex opens new avenues for therapeutic intervention. Targeting lipid metabolism, particularly OA pathways, could offer a strategy to modulate YAP activity and suppress tumour progression. In cancers where YAP is hyperactivated, lipid-based therapies may restore Hippo pathway function and inhibit oncogenic transcription. Additionally, disrupting ECM and cytoskeleton interactions via talin or PDLIM7 may impair mechanotransduction and reduce metastatic potential.

While the connection between the ECM, lipid signalling, and the Hippo signalling pathway regulation is compelling, the precise biochemical mechanism by which OA and PA influence complex formation and YAP phosphorylation remains insufficiently defined. Although the data show that OA disrupts interactions between YAP and ECM-associated proteins such as talin and CD2AP, the molecular basis for this disruption is unclear. It is possible that OA alters membrane fluidity or lipid raft composition, thereby affecting the localization or stability of scaffold proteins like ezrin and CD2AP. Alternatively, OA may induce post-translational modifications—such as phosphorylation or palmitoylation—of talin or PDLIM7, which could interfere with their ability to bind YAP. Another possibility is that OA indirectly activates upstream Hippo kinases, such as LATS1/2, through secondary signalling pathways, leading to increased YAP phosphorylation and cytoplasmic retention. In contrast, PA may promote YAP activation by inhibiting Hippo pathway regulators or enhancing ECM stiffness, which is known to drive mechanotransduction and nuclear YAP localization. To address these mechanistic gaps, future studies should incorporate lipidomic profiling: mass spectrometry to detect protein modifications such as SILAC, and live-cell imaging to monitor YAP dynamics in real time. Kinase activity assays and structural modelling of protein–lipid interactions would also provide valuable insights.

## 5. Conclusions

This study suggests a novel mechanistic link between lipid metabolism, extracellular matrix (ECM) signalling, and the Hippo pathway, with YAP serving as a potential central effector. Through a combination of in vitro and in vivo approaches, we demonstrate that oleic acid (OA) and palmitic acid (PA) affect YAP activity, with OA promoting YAP phosphorylation and inactivation in colorectal cancer cells. This potential lipid-induced regulation seems to be mediated through a newly identified and novel ECM-associated protein complex involving both ECM and cytoskeletal components talin, CD2AP, ezrin, and PDLIM7, which is disrupted upon OA treatment. The disruption of this complex impairs cell migration and alters transcriptional output, highlighting the convergence of mechanical and metabolic cues in controlling YAP function.

Importantly, our findings are replicated in vivo using the planarian model system, where OA treatment similarly suppresses Yorkie (YAP orthologue) activity and delays wound healing, reinforcing the conserved nature of the lipid/Hippo pathway regulation in a model organism. These results provide some evidence that lipid signalling may influence regenerative and oncogenic processes through the modulation of YAP and its associated protein networks.

While the study offers new insights into lipid/YAP regulation, several questions remain. The precise biochemical mechanisms by which OA and PA alter complex formation and kinase activity are not yet fully understood. Additionally, the use of supraphysiological lipid concentrations and cell line-specific responses highlights the need for broader validation across cancer types and under more physiologically relevant conditions.

Future research should focus on dissecting the molecular interactions within the ECM/YAP complex, exploring lipid-specific signalling pathways, and integrating these findings into models of cancer progression and tissue regeneration, specifically in more complex systems such as murine models. The use of assays looking at the links between direct effects from lipids on proteins may be required. Therapeutically, targeting lipid metabolism or disrupting ECM–YAP interactions may offer promising strategies to modulate YAP activity in cancer and fibrotic diseases. Overall, this work lays out the foundation for a deeper understanding of how metabolic and mechanical signals intersect to regulate cell fate and function.

## Data Availability

The original contributions presented in this study are included in the article. Further inquiries can be directed at the corresponding authors.

## Supplementary Materials

The following supporting information can be downloaded at: https://www.mdpi.com/article/10.3390/cells14211701/s1, Figure S1. Mass spectrometry–based analysis of YAP-interacting proteins regulated by lipid metabolism. Heatmap displaying changes in YAP-associated protein interactions identified by mass spectrometry following OA treatment. Each row represents a YAP-binding protein, with red indicating increased association (positive enrichment) and blue indicating decreased association (negative enrichment) relative to control conditions. Association is measured relative to binding to unique peptides when compared to control. The data highlight dynamic modulation of the YAP interactome in response to lipid metabolic cues.
