# Supplementary material for: The Regulation of the Hippo Signalling Pathway Effector YAP Through a Novel Lipid-Dependent Extracellular Matrix Complex
Source: Cells. 2025 Oct 30;14(21):1701. doi: 10.3390/cells14211701 (PMC12607357; doi:10.3390/cells14211701)
Supplement: Supplementary file 1 [file cells-14-01701-s001.zip › cells-3922931-supplementary.pdf]

Control	YAP IP	YAP IP + Oleic Acid	Uniprot Accession No	Protein name	Gene name
NEGATIVE	POSITIVE	POSITIVE	P13647	Keratin, type II cytoskeletal 5	KRT5
NEGATIVE	POSITIVE	POSITIVE	P46937	Transcriptional coactivator YAP1	YAP1
NEGATIVE	POSITIVE	POSITIVE	P02533	Keratin, type I cytoskeletal 14	KRT14
NEGATIVE	POSITIVE	NEGATIVE	Q13123	Protein Red	IK
NEGATIVE	POSITIVE	NEGATIVE	P15311	Ezrin	EZR
NEGATIVE	POSITIVE	POSITIVE	P14923	Junction plakoglobin	JUP
NEGATIVE	POSITIVE	NEGATIVE	P23588	Eukaryotic translation initiation factor 4B	EIF4B
NEGATIVE	POSITIVE	POSITIVE	Q86YZ3	Hornerin	HRNR
NEGATIVE	POSITIVE	NEGATIVE	P35241	Radixin	RDX
NEGATIVE	POSITIVE	NEGATIVE	P33993	DNA replication licensing factor MCM7	MCM7
NEGATIVE	POSITIVE	POSITIVE	P40227	T-complex protein 1 subunit zeta	CCT6A
NEGATIVE	POSITIVE	NEGATIVE	Q15061	WD repeat-containing protein 43	WDR43
NEGATIVE	POSITIVE	POSITIVE	O95429	BAG family molecular chaperone regulator	BAG4
NEGATIVE	POSITIVE	NEGATIVE	P54886	Delta-1-pyrroline-5-carboxylate synthase	ALDH18A1
NEGATIVE	POSITIVE	POSITIVE	Q9BZZ5	Apoptosis inhibitor 5	API5
NEGATIVE	POSITIVE	NEGATIVE	O95453	Poly(A)-specific ribonuclease PARN	PARN
NEGATIVE	POSITIVE	POSITIVE	P43243	Matrin-3	MATR3
NEGATIVE	POSITIVE	POSITIVE	P16401	Histone H1.5	H1-5
NEGATIVE	POSITIVE	POSITIVE	P48444	Coatomer subunit delta	ARCN1
NEGATIVE	POSITIVE	NEGATIVE	Q9H8H0	Nucleolar protein 11	NOL11
NEGATIVE	POSITIVE	POSITIVE	P16403	Histone H1.2	H1-2
NEGATIVE	POSITIVE	NEGATIVE	Q9Y3X0	Coiled-coil domain-containing protein 9	CCDC9
NEGATIVE	POSITIVE	POSITIVE	Q9NYF8	Bcl-2-associated transcription factor 1	BCLAF1
NEGATIVE	POSITIVE	NEGATIVE	Q9Y5Q8	General transcription factor 3C polypeptide 5	GTF3C5
NEGATIVE	NEGATIVE	POSITIVE	Q8WVV9	Heterogeneous nuclear ribonucleoprotein L-like	HNRNPLL
NEGATIVE	POSITIVE	POSITIVE	Q9UBD5	Origin recognition complex subunit 3	ORC3
NEGATIVE	POSITIVE	NEGATIVE	Q86XZ4	Spermatogenesis-associated serine-rich protein 2	SPATS2
NEGATIVE	POSITIVE	NEGATIVE	Q8WWY3	U4/U6 small nuclear ribonucleoprotein Prp31	PRPF31
NEGATIVE	POSITIVE	POSITIVE	P07477	Serine protease 1	PRSS1
NEGATIVE	POSITIVE	POSITIVE	P50990	T-complex protein 1 subunit theta	CCT8
NEGATIVE	POSITIVE	NEGATIVE	Q9NVP1	ATP-dependent RNA helicase DDX18	DDX18
NEGATIVE	POSITIVE	POSITIVE	Q99829	Copine-1	CPNE1
NEGATIVE	POSITIVE	NEGATIVE	Q06330	Recombining binding protein suppressor of hairless	RBPJ
NEGATIVE	POSITIVE	NEGATIVE	P42166	Lamina-associated polypeptide 2, isoform alpha	TMPO
NEGATIVE	POSITIVE	POSITIVE	Q9P2K5	Myelin expression factor 2	MYEF2
NEGATIVE	POSITIVE	POSITIVE	Q9Y2J4	Angiomotin-like protein 2	AMOTL2
NEGATIVE	POSITIVE	NEGATIVE	P06748	Nucleophosmin	NPM1

(blue)	NEGATIVE
(red)	POSITIVE

Figure S1. Mass spectrometry–based analysis of YAP-interacting proteins regulated by lipid metabolism. Heatmap displaying changes in YAP-associated protein interactions identified by mass spectrometry following OA treatment. Each row represents a YAP-binding protein, with red indicating increased association (positive enrichment) and blue indicating decreased association

(negative enrichment) relative to control conditions. Association is measured relative to binding to unique peptides when compared to control. The data highlight dynamic modulation of the YAP interactome in response to lipid metabolic cues.
